# A110 SUSTAINABILITY IN EVERYDAY GI PRACTICE: QUANTIFYING TRAVEL RELATED CARBON EMISSIONS FOR IN-PERSON APPOINTMENTS AND MODELS FOR REDUCTION THROUGH USE OF TELEHEALTH

**DOI:** 10.1093/jcag/gwae059.110

**Published:** 2025-02-10

**Authors:** C Galts, S Anvari, G Leontiadis

**Affiliations:** Gastroenterology, McMaster University, Hamilton, ON, Canada; Gastroenterology, McMaster University, Hamilton, ON, Canada; Gastroenterology, McMaster University, Hamilton, ON, Canada

## Abstract

**Background:**

The health care industry alone contributes to approximately 5% of annual carbon emissions and gastroenterology represent a more resource intensive subspecialty so much so that multiple societies have begun initiatives with aims to reduce the environmental impact of gastroenterology practice. After COVID-19 there was a large adoption of telehealth across many disciplines including gastroenterology. This had the benefit of reducing viral transmission but also significantly reduced travel related emissions for routine appointments. Since that time there has been a varied continuation of virtual care.

**Aims:**

We aimed to quantify the average carbon emissions associated with travel to non-endscopic gastroenterology appointments. We also aimed to develop models for emission reductions based on possible changes to practice (e.g. conversion of follow-up appointments to telehealth).

**Methods:**

Over a 2-week period, we conducted a cross-sectional analysis evaluating carbon emissions associated with travel to gastroenterology appointments. The average number of appointments per day was determined and by using postal codes we were able to estimate travel distances for patients. Carbon emissions were based on these travel distances using standard estimates (including average emissions by car, percentage of patients using alternate transportation, non-tail pipe emissions) and we assessed various estimated of emissions related to telehealth. We then used variable practice models to determine the potential emissions reductions.

**Results:**

We assessed 975 appointments, of which 71 were excluded (e.g. insufficient data, non-physician appointments), leaving 904 included appointments of which 75% were follow-up (678) and the remained were new consultations (226). Sixteen different gastroenterologists had an average of 22.7 patients per day. The mean return distance travelled per appointment was 57.3 km which translates to 14.9 kg CO2 per patient visit. An average day at in clinic would then equate to 337.3 kg CO2 per day, equivalent to 146.6 L gasoline or 15.5 trees’ annual carbon capture. By converting only appointments with a return distance over 100 km or follow-up appointments, we found that a 77% emissions reduction could be achieved.

**Conclusions:**

There can be significant emissions savings with conversion of in-person visits to telehealth while still allowing for some visits to be in-person. Given the unique nature of gastroenterology requiring in-person visits for endoscopy, this may serve as one mechanism by which the collective group of Canadian gastroenterologists can substantilally reduce carbon emissions related to their practice.

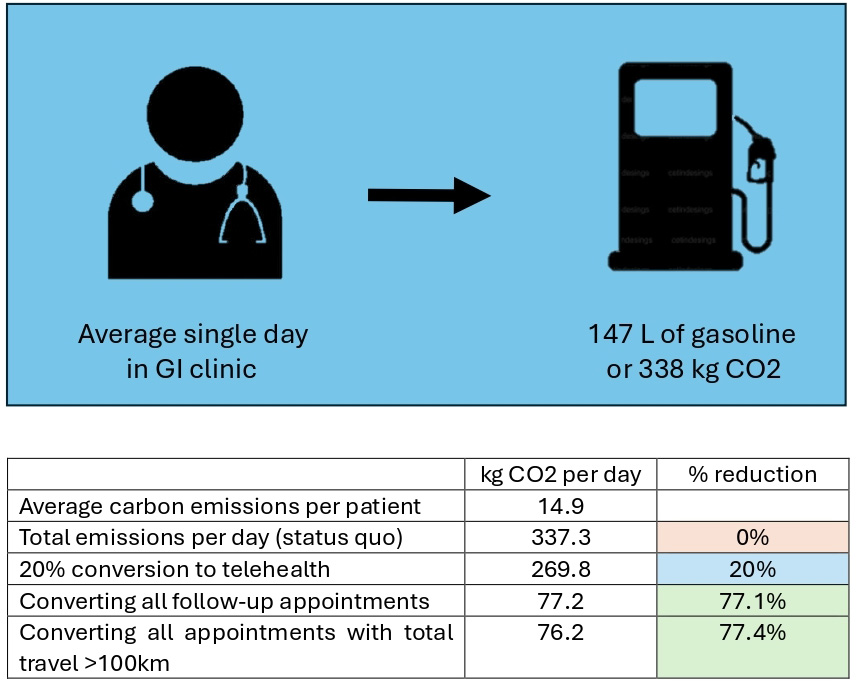

Impact on carbon emissions from conversion of in-person appointments to telehealth for gastroenterology

**Funding Agencies:**

None

